# MEF2C ameliorates learning, memory, and molecular pathological changes in Alzheimer’s disease *in vivo* and *in vitro*: Neuroprotective effects of MEF2C

**DOI:** 10.3724/abbs.2021012

**Published:** 2021-12-28

**Authors:** Jiamou Ren, Shuli Zhang, Xiaoling Wang, Yuxin Deng, Yi Zhao, Yan Xiao, Jian Liu, Liangzhao Chu, Xiaolan Qi

**Affiliations:** 1 Key Laboratory of Endemic and Ethnic Diseases Ministry of Education & Key Laboratory of Medical Molecular Biology of Guizhou Province Guizhou Medical University Guiyang 550004 China; 2 Department of Laboratory Medicine the 4th People′s Hospital of Guiyang Guiyang 550004 China; 3 Translational Medicine Research Center Guizhou Medical University Guiyang 550004 China; 4 Department of Neurosurgery Affiliated Hospital of Guizhou Medical University Guiyang 550004 China; 5 Chinese People′s Liberation Army Secret Service Center Sanatorium of Xiamen Xiamen 361000 China

**Keywords:** myocyte enhancer factor 2C (MEF2C), Alzheimer’s disease, APPswe/PSEN1dE9 double transgenic mice, oxidative stress, Nrf2-ARE

## Abstract

Myocyte enhancer factor 2C (MEF2C) is highly expressed in the nervous system, and regulates neuro-development, synaptic plasticity, and inflammation. However, its mechanism in Alzheimer’s disease (AD) is underestimated. In this study, the role and mechanism of MEF2C were investigated in the brain tissue specimens from patients with AD, APPswe/PSEN1dE9 double transgenic (APP/PS1_DT) mice, and SH-SY5Y cells treated with β-amyloid peptide (Aβ). The results indicated that the expression of MEF2C is significantly reduced, and the expression of MEF2C/Aβ in different parts of brain is negatively correlated in patients with AD. Knockdown of MEF2C promotes cell apoptosis and the level of β-amyloid precursor protein cleaving enzyme 1 (BACE) but reduces BACE2 expression. In addition, knockdown of *Mef2c*enhances the generation and aggregation of Aβ in the cortex of APP/PS1_DT mice, reduces the expression of synaptic proteins, exacerbates the ability of learning and memory of APP/PS1_DT mice, damages the structure of mitochondria, increases the oxidative stress (OS) level, and inhibits the expression levels of members of the Nrf2-ARE signal pathway. In summary, inhibition of MEF2C exacerbates the toxic effect of Aβ *in vivo* and *in vitro*, damages synaptic plasticity, reduces the ability of learning and memory of APP/PS1 mice, and increases the level of OS via the Nrf2-ARE signal pathway.

## Introduction

Alzheimer’s disease (AD) is a degenerative disease of the central nervous system characterized by progressive dementia and also the most common type of senile dementia [1]. The common neuropathological changes in AD are the formation of extracellular senile plaques, intracellular neurofibrillary tangles, neuronal loss, and brain atrophy [2]. The accumulating data have suggested that abnormal aggregation of β-amyloid peptide (Aβ), hyperphosphorylation of Tau protein, increased level of oxidative stress (OS) and inflammation are closely associated with the pathogenesis of AD [3]. In the amyloid toxicity hypothesis, it is considered that Aβ originates from the abnormal metabolism of amyloid precursor protein (APP) [4]. Aβ is released from APP by β-amyloid precursor protein cleaving enzyme 1 (BACE1) and γ-secretase. Previous studies have shown that soluble Aβ aggregates in cells to form Aβ oligomers (AβOs), which can cause damage to nerve cells in the brain [5]. Therefore, reducing the deposition of Aβ or attenuating its neurotoxicity is promising fundamental strategies for AD treatment.

A genome-wide association study showed that multiple factors, including myocyte enhancer factor 2C (MEF2C), have a strong correlation with the pathogenesis of AD [6]. *MEF2C* is mainly expressed in the cerebral cortex of animals [7], and has been identified as a susceptible gene for numerous neurological diseases by human genetic analysis. MEF2C can regulate the development and survival of neurons, as well as the formation of synapses in animal brains [ 8, 9]. It has also been confirmed that MEF2C is necessary for neuron survival. This effect is mainly caused by the activation of MEF2C from extracellular signal regulated kinase 5 (ERK5) [ 10, 11]; continuously activated MEF2C can rescue neuronal apoptosis mediated by NMDA receptors [12]; deletion of *Mef2c* in the brains of mice could damage hippocampus-dependent learning and memory [13]. Behavioral tests performed on adult mice with *Mef2c* knockdown in the brain showed that they had abnormal anxiety-like behaviors and decreased spatial cognitive function [14]. Although MEF2C plays an important role in the nervous system, there are few reports on the role of MEF2C in the pathogenesis of AD. The mRNA level of *MEF2C* in the blood of AD patients is lower than that of the normal control group, but the changes in the brain are not clear [15]. Through Kyoto Encyclopedia of Genes and Genomes (KEGG) analysis, it is found that MEF2C exists in the upstream of nuclear factor erythroid 2-related factor 2 (Nrf2) signal transduction pathway, which also shows that MEF2C is closely related to the Nrf2 signal pathway regulation.

Aβ is an inducer of OS, which increases the Aβ production and deposition. Therefore, it forms a vicious cycle that accelerates AD progression, and OS is an early mechanism in the AD pathogenesis [16]. The reactive oxygen species (ROS) overproduction and the antioxidant defense reduction directly damage synaptic activity and neurotransmission, leading to cognitive dysfunction [17]. As a transcription factor, Nrf2 can regulate the antioxidant proteins and redox balance in eukaryotic cells. The Nrf2-ARE signal pathway is the most important anti-OS signal pathway in AD, and plays an important role in maintaining the balance of oxidation and anti-oxidation in the body [18]. The combination of Nrf2 and ARE regulates transcriptional activation of important antioxidant enzymes such as NADPH quinone dehydrogenase 1 (NQO1), superoxide dismutase (SOD), and heme oxygenase 1 (HO-1) [19]. Compared with those in age-matched control individuals, the Nrf2 level in AD hippocampus was significantly reduced and restricted to the cytoplasm [20], and the activities of Nrf2-mediated SOD and glutathione peroxidase in AD tissues were reduced [21]. Up-regulation of Nrf2 in the hippocampus of 9-month-old APPswe/PSEN1dE9 double transgenic (APP/PS1_DT) mice could ameliorate spatial learning and memory [22]. In addition, Nrf2 activators, such as curcumin [23], resveratrol [24] and β-hydroxybutyrate [25], could alleviate or prevent Aβ-mediated reduction of cognitive deficits, and OS toxicity could affect AD in animal models. Decreasing the expression of Nrf2 could cause increased loss of synapse-related proteins in the brain of SAMP8 AD model mice [26]. In addition, in the TgCRND8 model mice, activating the Nrf2-ARE pathway can attenuate the release of IL-6 and TNF-α from microglia, thereby reducing neuroinflammation [27], which means that Nrf2 may be a potential therapeutic target for AD.

The aim of the present study is to investigate the expression and distribution of MEF2C and Aβ in the brain of AD patients and APP/PS1_DT mice, and to investigate the effects of MEF2C on reducing the production and aggregation of Aβ, relieving the neurotoxic effects of Aβ, and improving the learning and memory abilities of APP/PS1_DT mice by regulating the Nrf2-ARE signal pathway.

## Materials and Methods

### Ethics

The present study was conducted in accordance with ethical standards, the Declaration of Helsinki, National and International Guidelines, and was approved by the Ethics Committee of Guizhou Medical University.

### Reagents and antibodies

The TUNEL detection kit was purchased from Yisheng Biotechnology (Shanghai, China). Lipofectamine™ 3000 transfection reagent was ordered from Thermo Fisher Scientific (Waltham, USA). Reactive oxygen species (ROS) detection kit was purchased from BestBio Biotechnology (Shanghai, China). The apoptosis detection kit was purchased from KeyGEN Biotech (Nanjing, China). The adeno-associated virus that used to silence the mouse *Mef2c* gene (named Ad-shRNA-Mef2c), and the negative control (named Ad-shRNA-NC) were constructed by Buffal Biological Technology (Wuhan, China). The RNA interference plasmids for human *MEF2C*gene (named GV248-shRNA-MEF2C) and the negative control (named GV248-shRNA-NC) were constructed by Genechem (Shanghai, China). The interference RNA fragments of mouse *Mef2c*and NC were 5′-GTGGAGACATTGAGAAAGATT-3′ and 5′-GTTCGGTACAGATGCCTTAAT-3′, respectively; and 5′-GCCACAACAGTTTGGTGTA-3′ and 5′-TTCTCCGAACGTGTCACGT-3′ for SH-SY5Y cell *MEF2C* RNA interfering and NC, respectively. The methane dicarboxylic aldehyde (MDA) content detection kit and SOD activity detection kit were provided by Nanjing Jiancheng Bioengineering Institute (Nanjing, China). Alexa Fluor™ 488 goat anti-rabbit IgG antibody and Cy3 ^®^ goat anti-mouse IgG were obtained from Thermo Fisher Scientific. PBS, citrate, goat serum, and the immunohistochemistry universal two-step and DAB color kits were from OriGene Technologies (Maryland, USA).

The rabbit anti-MEF2C polyclonal antibody (ab211493), rabbit anti-HO-1 polyclonal antibody (ab13248), rabbit anti-BAX polyclonal antibody (ab32503), rabbit anti-BCL-2 polyclonal antibody (ab182858), rabbit anti-SYN polyclonal antibody (ab32594) and rabbit anti-BACE1 polyclonal antibody (ab108394) were all purchased from Abcam (San Francisco, USA). In addition, rabbit anti-NRF2 polyclonal antibody (gtx103322), rabbit anti-NQO1 polyclonal antibody (gtx100235), rabbit anti-SOD2 polyclonal antibody (gtx630559), rabbit anti-PSD95 polyclonal antibody (gtx133091), rabbit anti-β-actin polyclonal antibody (gtx109639), rabbit anti-GAPDH polyclonal antibody (gtx100118) and rabbit anti-BACE2 polyclonal antibody (gtx113951) were purchased from GeneTex (Irvine USA). Mouse anti-Aβ(6E10) antibody (803001) was purchased from BioLegend (San Diego, USA), and horseradish peroxidase (HRP)-conjugated anti-mouse secondary antibody (7076) and HRP-conjugated anti-rabbit secondary antibody (7074) were purchased from Cell Signaling Technology (Boston, USA).

### Brain sample origin

Post-mortem brain samples from the Dutch Brain Bank (Amsterdam, The Netherlands) are well characterized in terms of specific clinical and neuropathological criteria. The temporal and frontal cortex hippocampus of 8 patients with AD and 8 controls was investigated (3 slides of brain tissue of each patient was analyzed). The mean age at death for these patients with AD was 81.5±7.1 years old, while the mean age of the control cases was 79.4±9.2 years old; in AD and controls, the PMI (interval between death and autopsy) was 5.1±1.0 and 8.0±3.4 h, respectively. Table 1 provides the relevant details for each individual donor.

**Table:** **Table1** Clinical and neuropathological characteristics of patients with AD and normal controls

Case	Gender	Age (years)	PMI (h)	Braak (NFT)	Braak (Aβ)
C1	M	65	14.4	0	0
C2	F	78	6.5	1	A
C3	M	78	6.9	1	A
C4	M	82	13.6	2	B
C5	F	88	5.7	2	A
C6	F	78	4.3	1	A
C7	F	83	7.8	2	B
C8	M	72	8.7	1	A
AD1	M	85	7.2	5	C
AD2	F	76	5.1	5	C
AD3	F	80	5.3	5	C
AD4	M	73	3.6	6	C
AD5	F	95	4.8	6	C
AD6	M	88	5.0	5	C
AD7	F	86	4.8	6	C
AD8	F	72	3.8	4	B

NFT: Braak’s classification of stages (0−6) of AD based on the distribution and amount of neurofibrillary tangles. Aβ: Braak’s classification of the stages (A−C) of AD based on amyloid plaques.

### Immunohistochemical analysis

Brain tissues were fixed with 4% paraformaldehyde (PFA) in PBS for 24 h. Briefly, 4% PFA-fixed and paraffin-embedded brain tissues were cut into 4-μm slices. The slices were then deparaffinized, rehydrated and incubated for 30 min in 3% H _2_O _2_. Subsequently, the slices were repaired by citrate buffer and then blocked with 5% normal goat serum for 30 min, followed by incubation overnight with anti-Aβ(6E10) and anti-MEF2C primary antibodies (1:100) in a moist chamber at 4℃. For the detection of monoclonal antibodies, a peroxidase-conjugated anti-mouse VECTASTAIN ^®^ ABC kit (Vector Laboratories, Maravai LifeSciences, Burlingame, USA) was used. Negative control slides were incubated with non-immune serum in stead of the primary antibodies. The images were captured under an Eclipse Ci-E microscope (Nikon, Tokyo, Japan) and analyzed using the NIS-Elements Advanced Research software (Nikon).

### APP/PS1_DT mice reproduction and treatment

The male breeder (20–30 g) of APP/PS1_DT mice (B6. Cg-Tg, referred as APP/PS1; animal license No. SCXK 2014-0002) was purchased from Shanghai Southern Model Biotechnology (Shanghai, China). The genotypes of these mice were further confirmed by PCR to insure the presence of the APPswe/PSldE9 gene mutations. The verification primers designed for APP and PS1 were: *APP* forward 5′-GACTGACCACTCGACCAGGTTCTG-3′, reverse 5′-CTTGTAAGTTGGATTCTCATATCCG-3′; *PS1*  forward 5′-AATAGAGAACGGCAGGAGCA-3′, reverse 5′-GCCATGAGGGCACTAATCAT-3′. The expected length of PCR products were 400 and 600 bp, respectively. APP/PS1 mice were subsequently referred to as APP/PS1 mice ( Supplementary Figure S1).

Different treatments were applied to APP/PS1_DT mice in the following groups (10 mice in each group). In the wild-type (WT)+Ad-shRNA-NC group, 6 and 10-month-old C57 WT mice were injected with adenovirus containing Ad-shRNA-NC in the lateral ventricle; in the WT+Ad-shRNA-Mef2c group, 6 and 10-month-old C57 WT mice were injected with adenovirus containing Ad-shRNA-Mef2c recombinant plasmid in the lateral ventricle; in the APP/PS1+Ad-shRNA-NC group, 6 and 10-month-old APP/PS1 mice were injected with adenovirus containing Ad-shRNA-NC in the lateral ventricle; in the APP/PS1+Ad-shRNA-Mef2c group, 6 and 10-month-old APP/PS1 mice were injected with adenovirus containing Ad-shRNA-Mef2c recombinant plasmid in the lateral ventricle. Two weeks after injection of the previously mentioned virus in the lateral ventricle, the silencing efficiency of *Mef2c* in the cortex of mice was detected by western blot analysis.

### Morris water maze (MWM) test

The Morris water maze method was used to detect the learning and memory abilities of the mice in each group. For the positioning navigation experiment, labyrinth was prepared as required: the platform was placed in quadrant IV, and all mice were placed into the water according to the midpoint of the quadrants of IV, III, II and I every day. This was continuously tested for 4 days, and the head was kept facing the upper side of the labyrinth wall for 60 s each time. If a mouse was not able to find the platform after 60 s, it would be guided to the platform, where it would stay for 10 s. The recording time was 60 s. For the space exploration experiment, the platform was removed on day 5, and the number of times that the mice searched for platforms in each quadrant and the length of the stay in the flat within 60 s were recorded. The number of times that the platform was found and the length of stay on the platform indicate the memory ability.

### Immunofluorescence analysis

The right side of the mouse brain was collected, fixed with 4% PFA for 24 h, dehydrated, paraffin embedded and sectioned. The brain slides of AD patients and mice were then dewaxed, citrate repaired, blocked with sheep serum solution and incubated with the corresponding primary antibody at 4℃ overnight. After the primary antibody was discarded, the corresponding diluted secondary antibody (Cy3 ^®^ labeled anti-mouse IgG and Alexa Flour™ 488-labeled anti-rabbit IgG) was added dropwise, and the plate was sealed with VECTASHIELD ^®^ after 1 h of incubation at room temperature. The images were observed and collected using a fluorescence inverted microscope (Nikon), and at least two fields of view were randomly selected for each group to be photographed for 200 times. The background light of each photograph was consistently maintained. Image-Pro Plus 6.0 software was used to analyze the green and red fluorescence of each image to obtain its integrated optical density (IOD).

### TUNEL staining

The right side of the mouse brain was collected (including the cortex), fixed with 4% PFA for 24 h, dehydrated, paraffin embedded and sectioned. The sections were then dewaxed, and reagents were added according to the instructions of the manufacturer of the TUNEL detection kit (cat. no. 40308ES20; Shanghai Yisheng Biotechnology). Finally, DAPI was added dropwise and incubated for 5 min in the dark to stain the specimen. Next, the excess DAPI was rinsed away with PBST four times (5 min each). The liquid on the section was then dried with absorbent paper, and the section was mounted with a mounting solution containing an anti-fluorescence quencher. The images were observed and collected under a BX53 fluorescence microscope (Olympus, Tokyo, Japan). The apoptotic cells were red, while blue represented the nucleus.

### Detection of MDA content and SOD activity

Cerebral cortex tissue (0.15 g) was placed in an EPP tube, and supplemented with 1.5 mL of saline, followed by homogenization on ice with a homogenizer and centrifugation at 793.75 *g* for 10 min. The supernatant was then collected and used to prepare a brain tissue homogenate. The MDA content and SOD activity were assayed and calculated using the corresponding commercial kits in accordance with the manufacturer’s instructions (MDA: A003-1; SOD: A001; Nanjing Jiancheng Bioengineering Institute).

### Western blot analysis

Total protein concentration was measured by Coomassie Brilliant Blue protein assay. The proteins were separated by 10% SDS-PAGE and transferred to polyvinylidene difluoride (PVDF) membranes (Merck, Millipore, USA). After being blocked with western blocking buffer (Beyotime Biotechnology) for 2 h, the membranes were incubated with primary antibodies against the indicated proteins. The membranes were then rinsed and incubated with HRP-conjugated anti-mouse or rabbit secondary antibody for 60 min. Finally, the protein bands were visualized with an ECL detection system (GeneGnome XRQ NPC; Syngene). Subsequently, the membranes with antibodies were rinsed off, and the membranes were incubated with antibodies against β-actin (1:5000) or GAPDH (1:5000) for 120 min and then with HRP-conjugated anti-mouse or rabbit secondary antibody for 60 min. The expressions of the target proteins were calculated relative to the expression level of β-actin or GAPDH which was used as loading control.

### AβO preparation

Aβ was prepared according to a previously reported method [27]. Synthetic human Aβ _1-42_ was suspended in pre-cooled hexafluoroisopropanol (HFIP; Sigma) at a final concentration of 1 mM and then incubated at room temperature for 60 min, followed by incubation on ice for 10 min. Aliquots of Aβ solution were transferred into non-siliconized microcentrifuge tubes, and HFIP was allowed to evaporate overnight under a hood at room temperature, then stored at –80℃. Prior to treatment of the cells, Aβ _1-42_ was dissolved in DMSO to obtain a final concentration of 5 mM. For the preparation of oligomers, this solution was diluted with modified DMEM (Sigma) and then incubated for 24 h or at 4℃.

### Cell transfection

SH-SY5Y cells, a human neuroblastoma cell line purchased from the German Collection of Microorganisms and Cell Cultures (Braunschweig, Germany), were grown to 70% confluence and subject to cell transfection with GV248-shRNA-MEF2C or GV248-shRNA-NC using the Lip3000 kit (L3000015; Thermo Fisher Scientific) according to the manufacturer’s instructions. The transfection efficiency was observed by fluorescence microscopy (Nikon), and the silencing efficiency of MEF2C was detected by western blot analysis ( Supplementary Figure S3). The cells were treated as follows: Group A, GV248-shRNA-NC; group B, GV248-shRNA-MEF2C; group C, GV248-shRNA-NC+Aβ; and group D, GV248-shRNA-MEF2C+Aβ. For group C and D, 48 h after transfection, the cells were exposed to 1 μM AβO for 24 h. Cells were collected for subsequent experiments to detect cell apoptosis, ROS and protein levels.

### Flow cytometric analysis

The cell apoptosis rate of each group was detected using the Annexin V-APC/7-AAD Apoptosis Detection Kit (KGA1026; KeyGEN Biotech) according to the manufacturer’s instructions, and the ROS level was determined using the DHE-ROS detection kit (BB-47051; Shanghai BestBio Biotechnology) according to the manufacturer’s instructions. The treated cells were immediately analyzed by flow cytometry on the BD FACSVerse flow cytometer (Becton, Dickinson and Company, Franklin Lakes, USA).

### Statistical analysis

Statistical analysis was performed using SPSS 22.0, and data were presented as the mean±standard deviation. Statistical differences between two groups were determined by two-tailed Student’s *t*-test, while statistical differences between multiple groups were determined by one-way ANOVA. *P*<0.05 was considered to be statistically significant.

## Results

### Distribution of Aβ and MEF2C in the brain of AD patients

**Figure 1:**
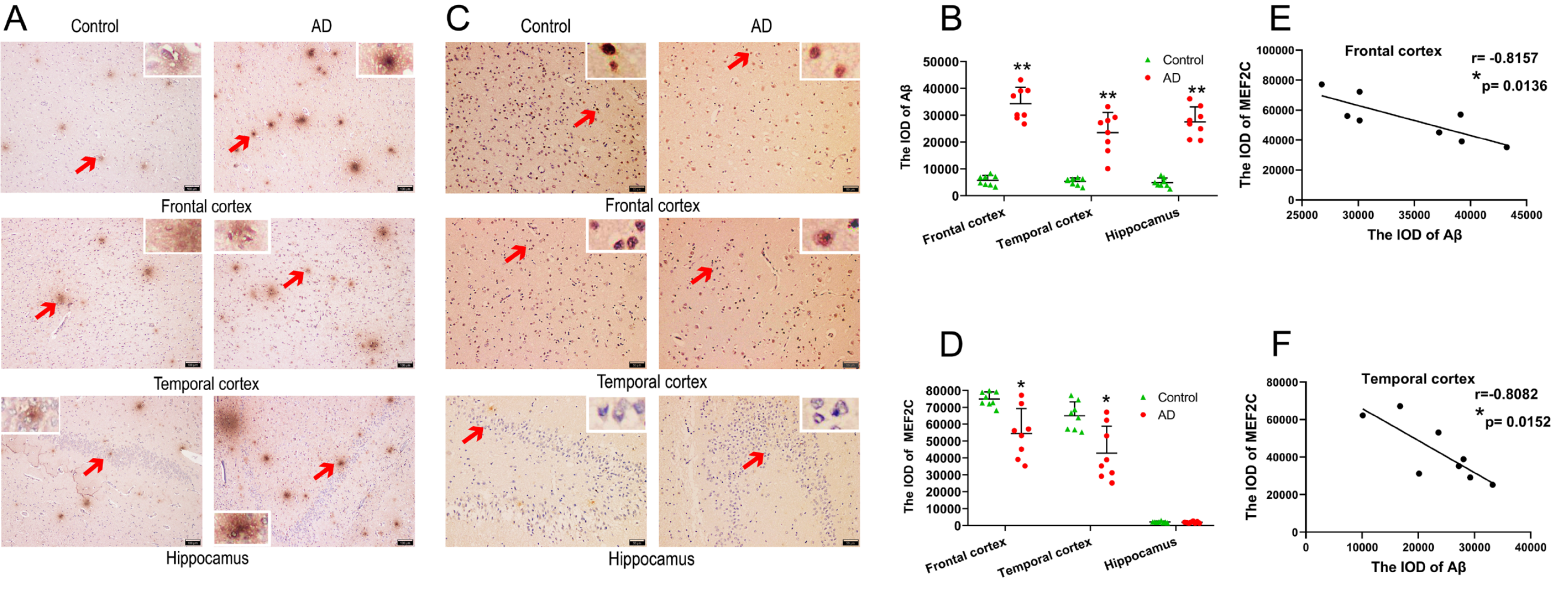
Figure1 **Distribution of Aβ and MEF2C and the correlation between the IOD levels of MEF2C and Aβ in the brains of patients with AD and normal controls**(A) Senile plaques were detected in the frontal lobe, temporal lobe and hippocampus of patients with AD and normal controls by immunohistochemistry. (B) Integrated optical density of Aβ in various brain regions of patients with AD and the controls. Arrows indicate senile plaque staining. Scale bar, 100 μm. (C) The expression of MEF2C was detected in the frontal lobe, temporal lobe and hippocampus of patients with AD and controls by immunohistochemistry. (D) Integrated optical density of MEF2C in various brain regions of patients with AD and controls. Scale bar, 50 μm. Data are presented as the mean±standard deviation. (E) Correlation between MEF2C and Aβ IOD values in frontal lobe. (F) Correlation between MEF2C and Aβ IOD values in temporal lobe. r refers to the relevance coefficient. *P<0.05 and **P<0.01 vs control.

In order to detect whether MEF2C expression was changed and analyze the correlation between MEF2C and Aβ levels, we used immunohistochemical analysis to detect the expressions of MEF2C and Aβ in the brains of AD patients. The results showed that extensive Aβ aggregation appeared in the temporal lobe, frontal lobe and hippocampus of patients with AD, while limited Aβ aggregation was observed in the control group ( Figure 1A). Compared with that of the control group, the area of senile plaques was significantly larger ( Figure 1B). MEF2C is scattered in the temporal and frontal lobes of patients with AD and normal controls, but not in the hippocampus ( Figure 1C). The immunohistochemical results showed that the expression of MEF2C in the temporal and frontal lobes of AD patients was lower than that of the normal controls, while MEF2C was not expressed in the hippocampus ( Figure 1D). Pearson correlation analysis revealed that the IOD values of MEF2C and Aβ were negatively correlated in the frontal ( Figure 1E) and temporal ( Figure 1F) lobes of AD patients. These data indicate that the decrease of MEF2C may be due to the deposition of Aβ.

### Knockdown of Mef2c increased Aβ deposition in the cortex of APP/PS1_DT mice

**Figure 2:**
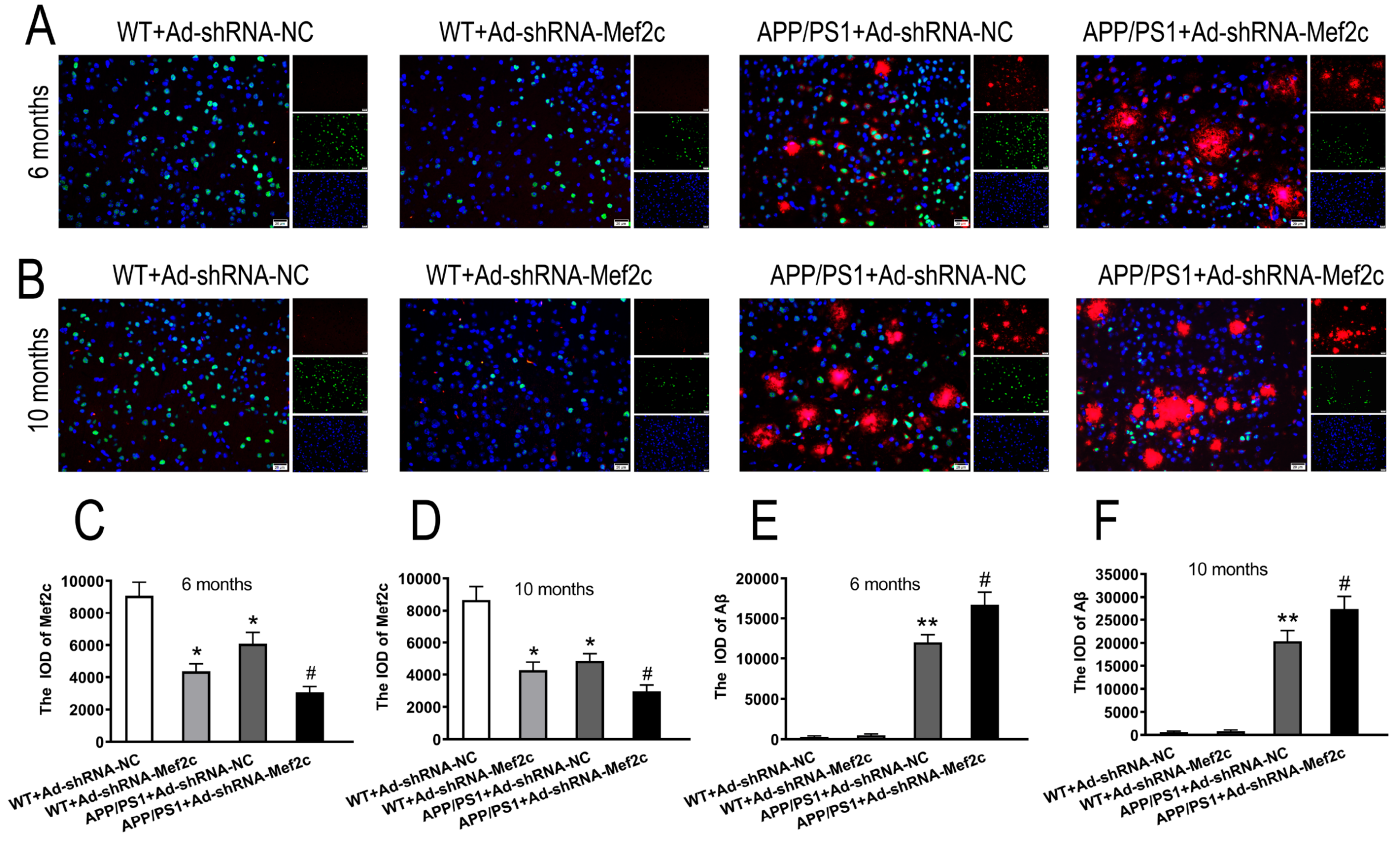
Figure2 **Knockdown of Mef2c increased the deposition of Aβ in the cortex of APP/PS1 mice**(A,B) Immunofluorescence dual labeling of Mef2c and Aβ in the cortex of mice. (C,D) IOD of Mef2c in the cortex of 6- and 10-month-old mice. (E,F) IOD of Aβ in the cortex of 6- and 10-month-old mice. C57 WT mice were injected with Ad-shRNA-NC (WT+Ad-shRNA-NC) or Ad-shRNA-Mef2c (WT+Ad-shRNA-Mef2c). APP/PS1 mice were injected with Ad-shRNA-NC (APP/PS1+Ad-shRNA-NC) or Ad-shRNA-Mef2c (APP/PS1+Ad-shRNA-Mef2c). Red represents a positive result for Aβ, while green represents a positive result for Mef2c. The cell nuclei were stained blue with DAPI in panels A and B. Scale bar, 20 μm. Data are presented as the mean±standard deviation. Ad-shRNA, Ad-shRNA-NC. *P<0.05 vs WT+Ad-shRNA-NC; #P<0.05 vs APP/PS1+Ad-shRNA-NC.

To further study the changes of Mef2c and Aβ in the brain of APP/PS1_DT mice. We detected the levels and localization of Mef2c and Aβ, and the effects of Mef2c knockdown on the deposition of Aβ by immunofluorescence double labeling. The results showed that Mef2c was largely expressed in the cortex of mice, mainly in the nucleus, while Aβ was mainly expressed in the cytoplasm of APP/PS1 mice ( Figure 2A,B). Compared with that of the WT+Ad-shRNA-NC group, the expression of Mef2c was reduced in the WT+Ad-shRNA-Mef2c and APP/PS+Ad-shRNA-NC groups. Compared with that of the APP/PS1+Ad-shRNA-NC group, the protein level of Mef2c was reduced in the APP/PS1+Ad-shRNA-Mef2c group ( Supplementary Figure S2 and Figure 2C,D).

APP/PS1_DT mice showed a large quantity of Aβ aggregation, while WT mice exhibited no Aβ aggregation in the cortex ( Figure 2E, F). Compared with that of the APP/PS1+Ad-shRNA-NC group, the level of Aβ aggregation was increased in the APP/PS1+Ad-shRNA-Mef2c group ( Figure 2E,F).

These data further suggest that MEF2C and Aβ have a certain correlation in the pathogenesis of AD, and silencing MEF2C can increase the deposition of Aβ.

### Knockdown of Mef2c reduced the learning and memory abilities of APP/PS1_DT mice

**Figure 3:**
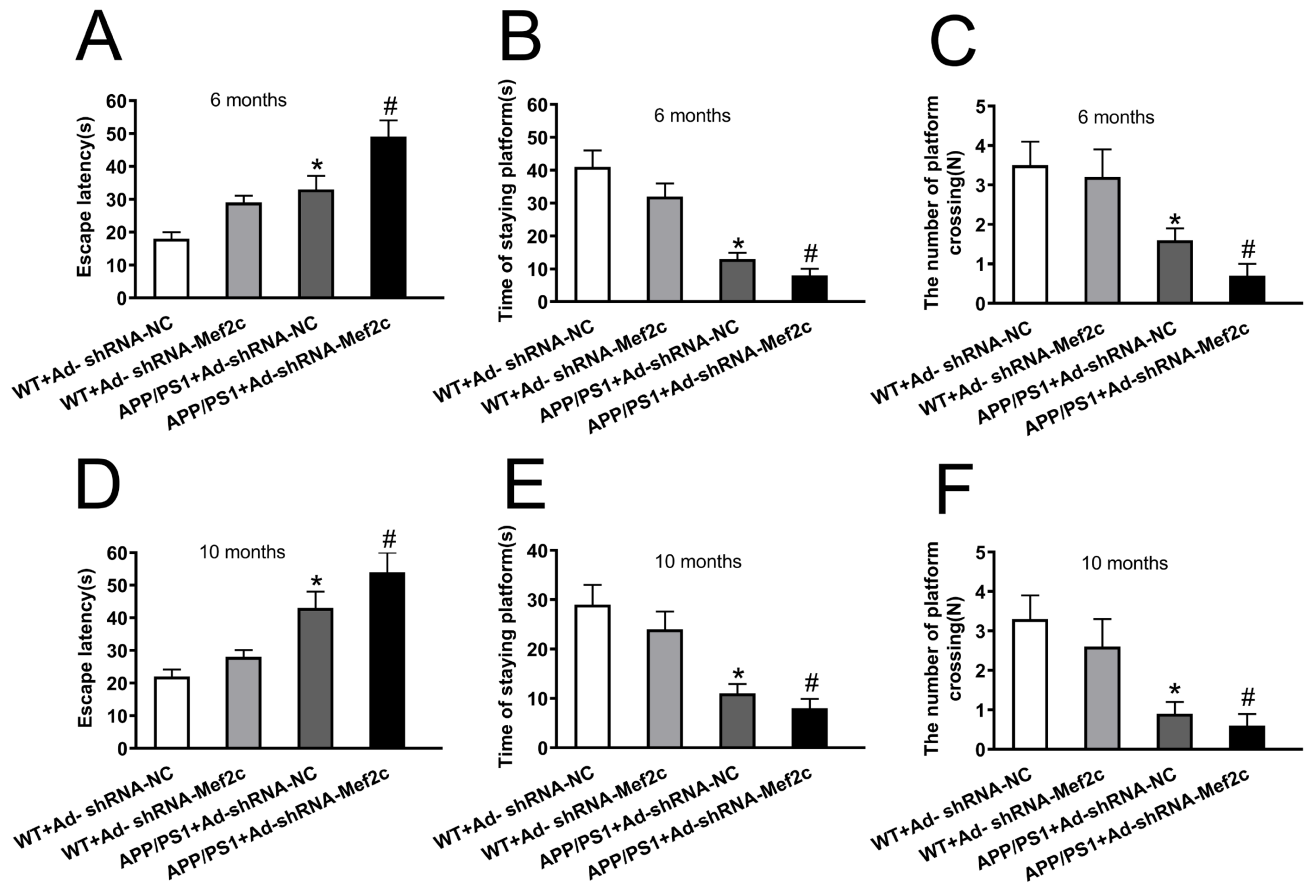
Figure3 **Knockdown of Mef2c reduced learning and memory abilities of APP/PS1 mice**(A,D) Escape latency of 6- and 10-month-old mice. (B,E) Platform stay time of 6- and 10-month-old mice. (C,F) Number of crossing platforms for 6- and 10-month-old mice. C57 WT mice were injected with Ad-shRNA-NC (WT+Ad-shRNA-NC). C57 WT mice were injected with Ad-shRNA-Mef2c (WT+Ad-shRNA-Mef2c). APP/PS1 mice were injected with Ad-shRNA-NC (APP/PS1+Ad-shRNA-NC). APP/PS1 mice were injected with Ad-shRNA-Mef2c (APP/PS1+Ad-shRNA-Mef2c). Data are presented as the mean±standard deviation. *P<0.05 vs WT+Ad-shRNA-NC; #P<0.05 vs APP/PS1+Ad-shRNA-NC.

To examine whether knockdown of Mef2c increases the cognitive deficits in APP/PS1_DT mice, we measured the potential of Mef2c to alleviate AD-induced declines in spatial memory and learning ability by using the MWM test. Compared with that of the WT+Ad-shRNA-NC group, the escape latency of the APP/PS1+Ad-shRNA-NC group was significantly longer; compared with that of mice in the APP/PS1+Ad-shRNA-NC group, the escape latency period of mice in the APP/PS1+Ad-shRNA-Mef2c group was prolonged ( Figure 3A,D). Compared with the WT+Ad-shRNA-NC mice, the APP/PS1+Ad-shRNA-NC mice stayed on the platform for a shorter time ( Figure 3B,E), and the number of crossing platforms was decreased ( Figure 3C,F). Compared with the findings in the APP/PS1+Ad-shRNA-NC group, the APP/PS1+Ad-shRNA-Mef2c group showed a shorter time on the platform ( Figure 3B,E), and a decreased number of crossing platforms ( Figure 3C,F). These results indicate that knockdown of Mef2c increased the spatial learning and memory disorders in APP/PS1_DT mice.

### Knockdown of Mef2c increased apoptosis in cortex of APP/PS1_DT mice and SH-SY5Y cells treated with AβO

**Figure 4:**
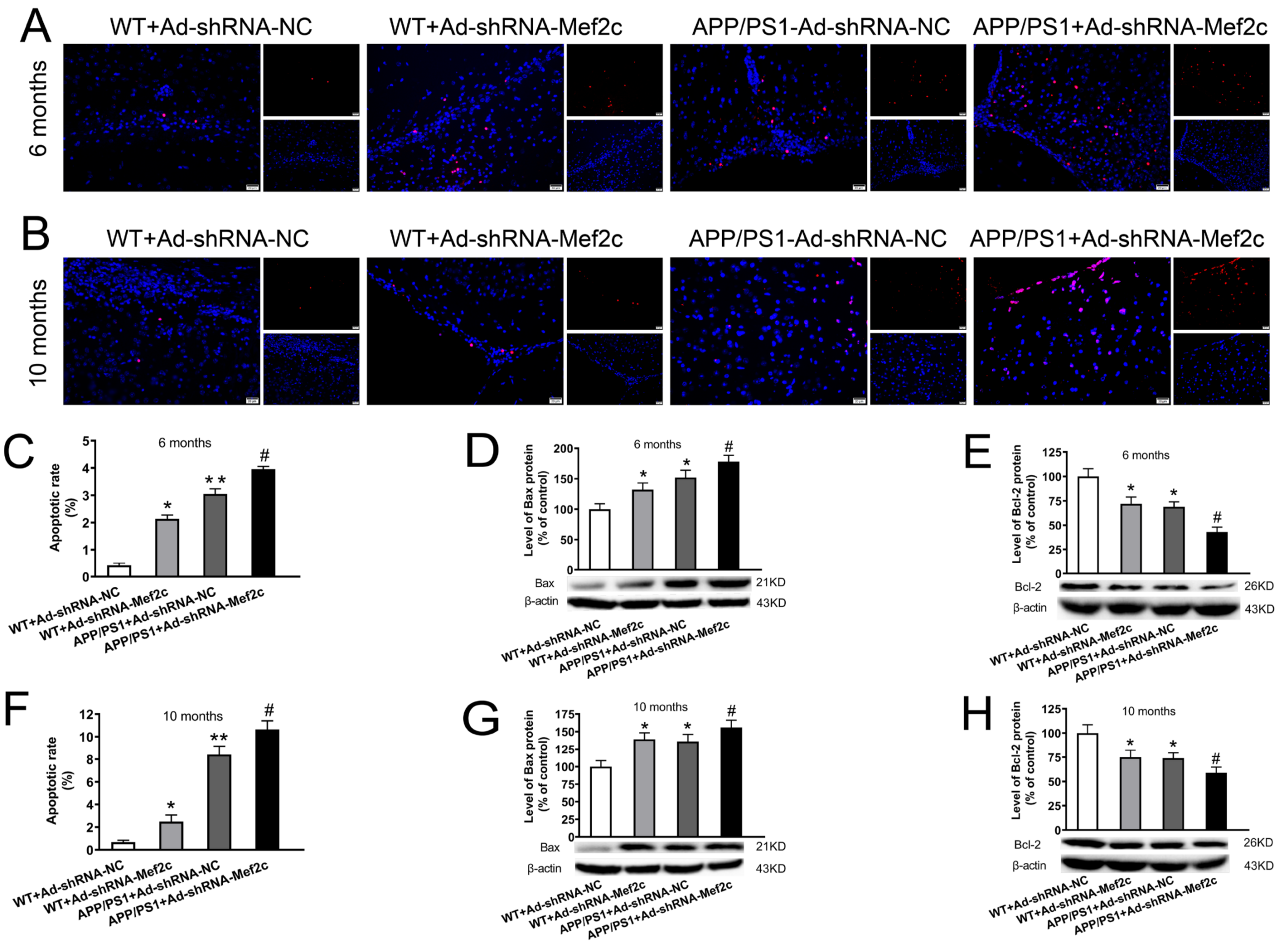
Figure4 **Knockdown of Mef2c increased apoptosis in the cortex of APP/PS1 mice**(A,B) The level of apoptosis in the cortex of 6- and 10-month-old mice was detected by TUNEL assay. Red represents cells undergoing apoptosis, while blue represents the cell nuclei which were stained with DAPI. (C,F) Apoptosis rate in 6- and 10-month-old mice. (D,G) The level of BAX in the cortex of 6- and 10-month-old mice was detected by western blot analysis. (E,H) The level of BCL-2 in the cortex of 6- and 10-month-old mice was detected by western blot analysis. C57 WT mice were injected with Ad-shRNA-NC (WT+Ad-shRNA-NC) or Ad-shRNA-Mef2c (WT+Ad-shRNA-Mef2c). APP/PS1 mice were injected with Ad-shRNA-NC (APP/PS1+Ad-shRNA-NC) or Ad-shRNA-Mef2c (APP/PS1+Ad-shRNA-Mef2c). Data are presented as the mean±standard deviation. *P<0.05, **P<0.01 vs WT+Ad-shRNA-NC; #P<0.05 vs APP/PS1+Ad-shRNA-NC.

**Figure 5:**
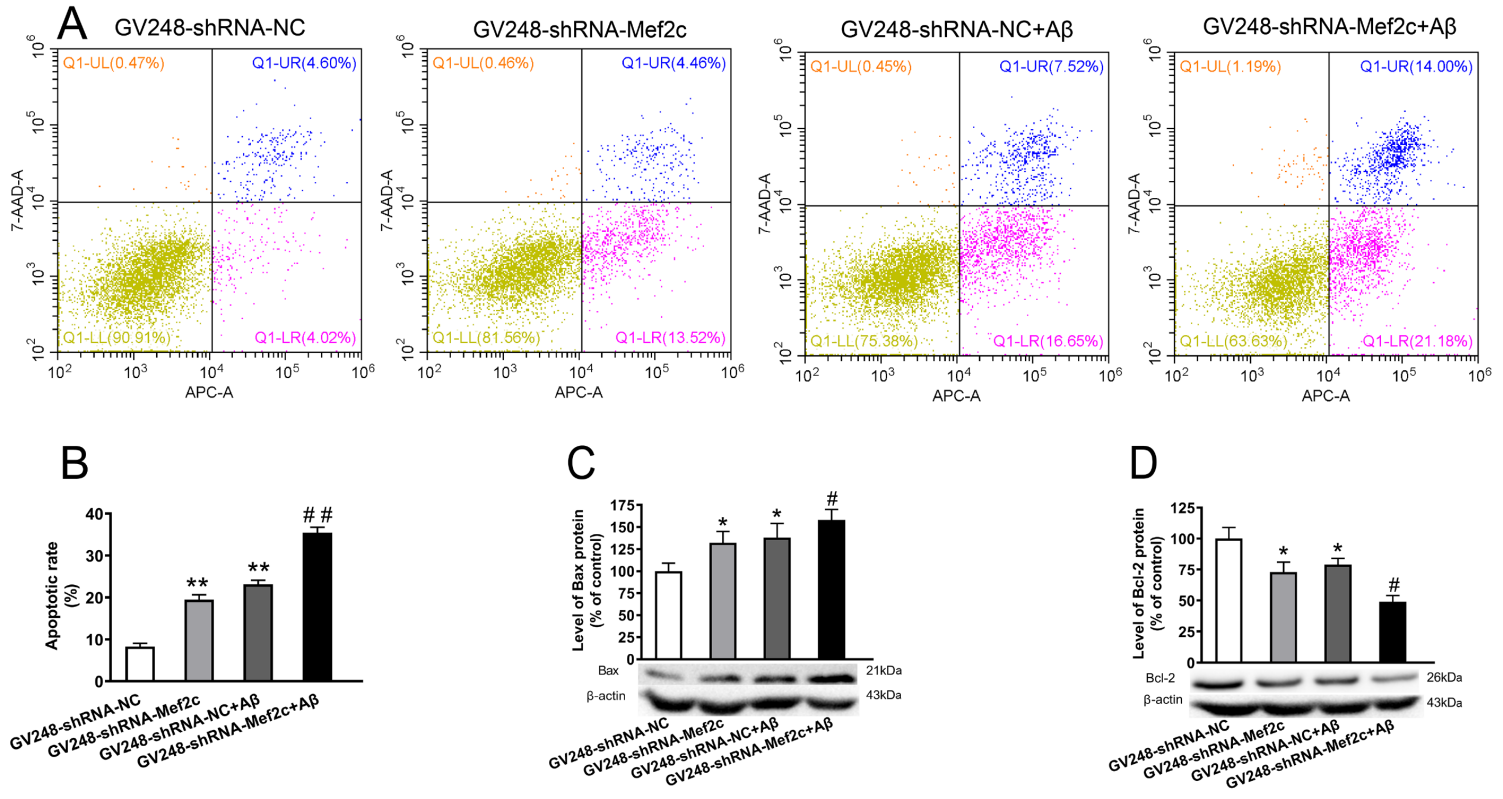
Figure5 **Knockdown of MEF2C increased apoptosis in SH-SY5Y cells treated with AβO**(A) The level of apoptosis in cells was detected by flow cytometry. (B) Apoptosis rate in each group of cells. (C) The protein level of BAX in cells was detected by western blot analysis. (D) The protein level of BCL-2 in cells was detected by western blot anaysis. SH-SY5Y cells were transfected with GV248-shRNA-NC (GV248-shRNA-NC) or GV248-shRNA-Mef2c (GV248-shRNA-Mef2c). SH-SY5Y cells were transfected with GV248-shRNA-NC and exposed to AβO (GV248-shRNA-NC+Aβ). SH-SY5Y cells were transfected with GV248-shRNA-Mef2c and exposed to AβO (GV248-shRNA-Mef2c+Aβ). Data are presented as the mean±standard deviation. *P<0.05, **P<0.01 vs GV248-shRNA-NC; #P<0.05, ##P<0.01 vs GV248-shRNA-NC+Aβ.

To test whether decreased level of Mef2c increases the apoptotic rate of neuronal cells in cortex of APP/PS1_DT mice and SH-SY5Y cells, TUNEL assay and flow cytometry were used and apoptosis-related proteins were detected by western blot analysis. Compared with that in the WT+Ad-shRNA-NC group, the apoptosis rate was increased in the WT+Ad-shRNA-Mef2c and APP/PS1+Ad-shRNA-NC group ( Figure 4A,B); compared with that in the APP/PS1+Ad-shRNA-NC group, the apoptosis rate was increased in the APP/PS1+Ad-shRNA-Mef2c group ( Figure 4C,F). Compared with that in the WT+Ad-shRNA-NC group, BAX protein expression was increased in the WT+Ad-shRNA-Mef2c and APP/PS1+Ad-shRNA-NC groups ( Figure 4D,G), while BCL-2 protein expression was decreased ( Figure 4E,H). Compared with that in the APP/PS1+Ad-shRNA-NC group, BAX protein expression was increased ( Figure 4D,G), while BCL-2 protein expression was decreased ( Figure 4E,H) in the APP/PS1+Ad-shRNA-Mef2c group. The results were similar in SH-SY5Y cells treated with AβO. The expression of MEF2C was decreased significantly with GV248-shRNA-MEF2C transfection ( Supplementary Figure S3). The apoptosis rates of AβO-treated SH-SY5Y cells and SH-SY5Y cells with MEF2C knockdown were significantly increased compare to that in the GV248-shRNA-NC group ( Figure 5A,B), with increased the protein level of Bax ( Figure 5C) and decreased the level of BCL-2 protein ( Figure 5D). These data indicate that MEF2C reduction may aggravate the neurotoxic effects of Aβ by increasing the rate of apoptosis.

### Knockdown of Mef2c in the cortex of APP/PS1_DT mice increased the expression of BACE1 and decreased the expression of BACE2

**Figure 6:**
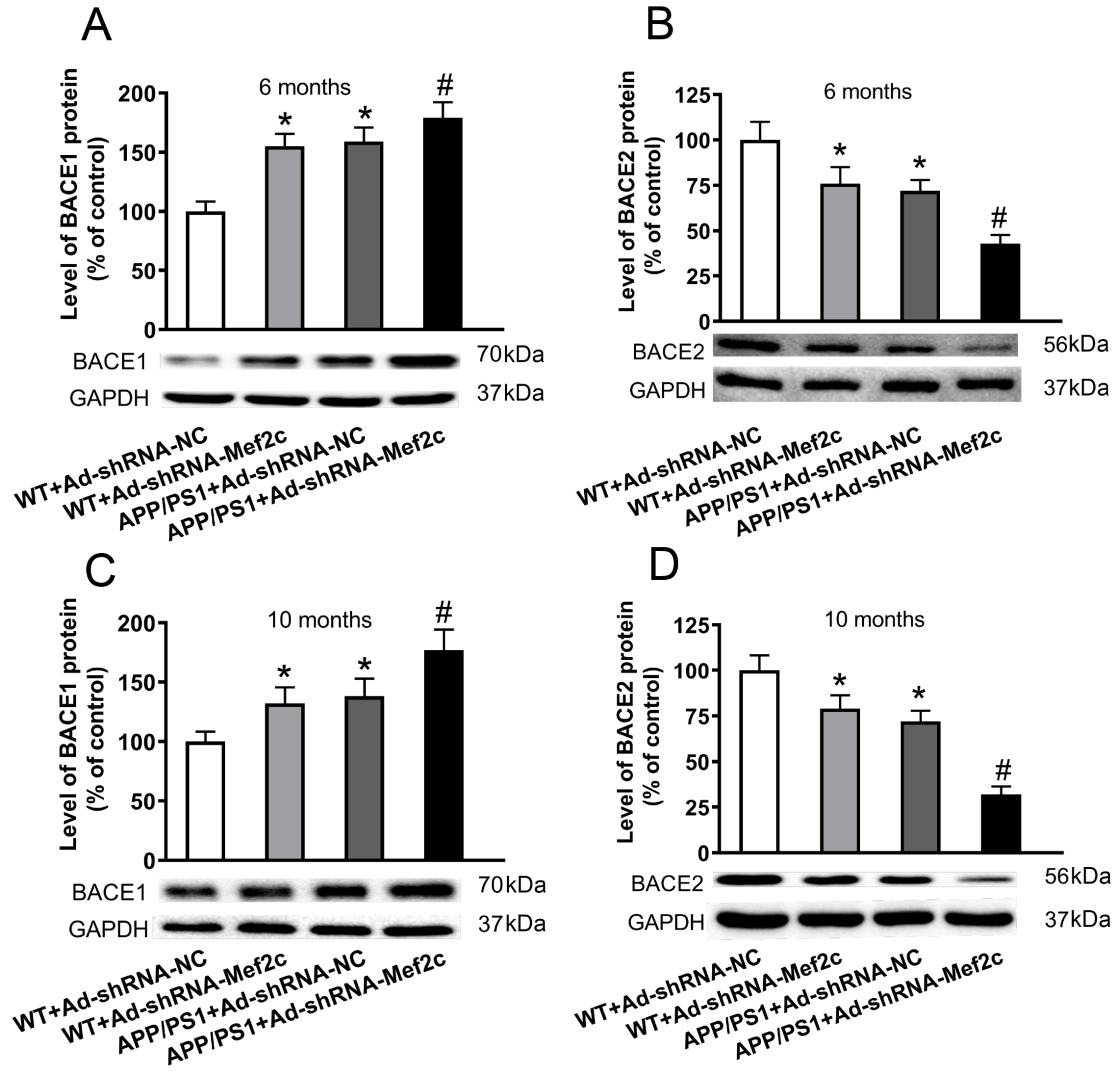
Figure6 **Knockdown of Mef2c in the cortex of APP/PS1 mice increased the expression of BACE1 and decreased the expression of BACE2**(A,C) The level of BACE1 in the cortex of 6- and 10-month-old mice was detected by western blot analysis. (B,D) The level of BACE2 in the cortex of 6- and 10-month-old mice was detected by western blot analysis. C57 WT mice were injected with Ad-shRNA-NC (WT+Ad-shRNA-NC) or Ad-shRNA-Mef2c (WT+Ad-shRNA-Mef2c). APP/PS1 mice were injected with Ad-shRNA-NC (APP/PS1+Ad-shRNA-NC) or Ad-shRNA-Mef2c (APP/PS1+Ad-shRNA-Mef2c). Data are presented as the mean ± standard deviation. *P<0.05 vs WT+Ad-shRNA-NC; #P<0.05 vs APP/PS1+Ad-shRNA-NC.

In order to detect the effect of Mef2c on APP metabolism in APP/PS1_DT mice, we detected the levels of key enzymes in APP metabolism by western blot analysis. The results showed that compared with that of the WT+Ad-shRNA-NC group, the expression of BACE1 protein was increased, while the expression of BACE2 protein was decreased in the WT+Ad-shRNA-Mef2c and APP/PS1+Ad-shRNA-NC groups ( Figure 6A,C). Compared with that of APP/PS1+Ad-shRNA-NC group, the expression of BACE1 protein was increased, while the expression of BACE2 protein was decreased in APP/PS1+Ad-shRNA-Mef2c group ( Figure 6B,D). These data indicate that Mef2c knockdown increases the production of Aβ by elevating the expression of BACE1 and reducing the expression of BACE2.

### Knockdown of Mef2c reduced the expressions of Nrf2-ARE pathway-related proteins in the cortex of APP/PS1_DT mice and SH-SY5Y cells treated with AβO

**Figure 7:**
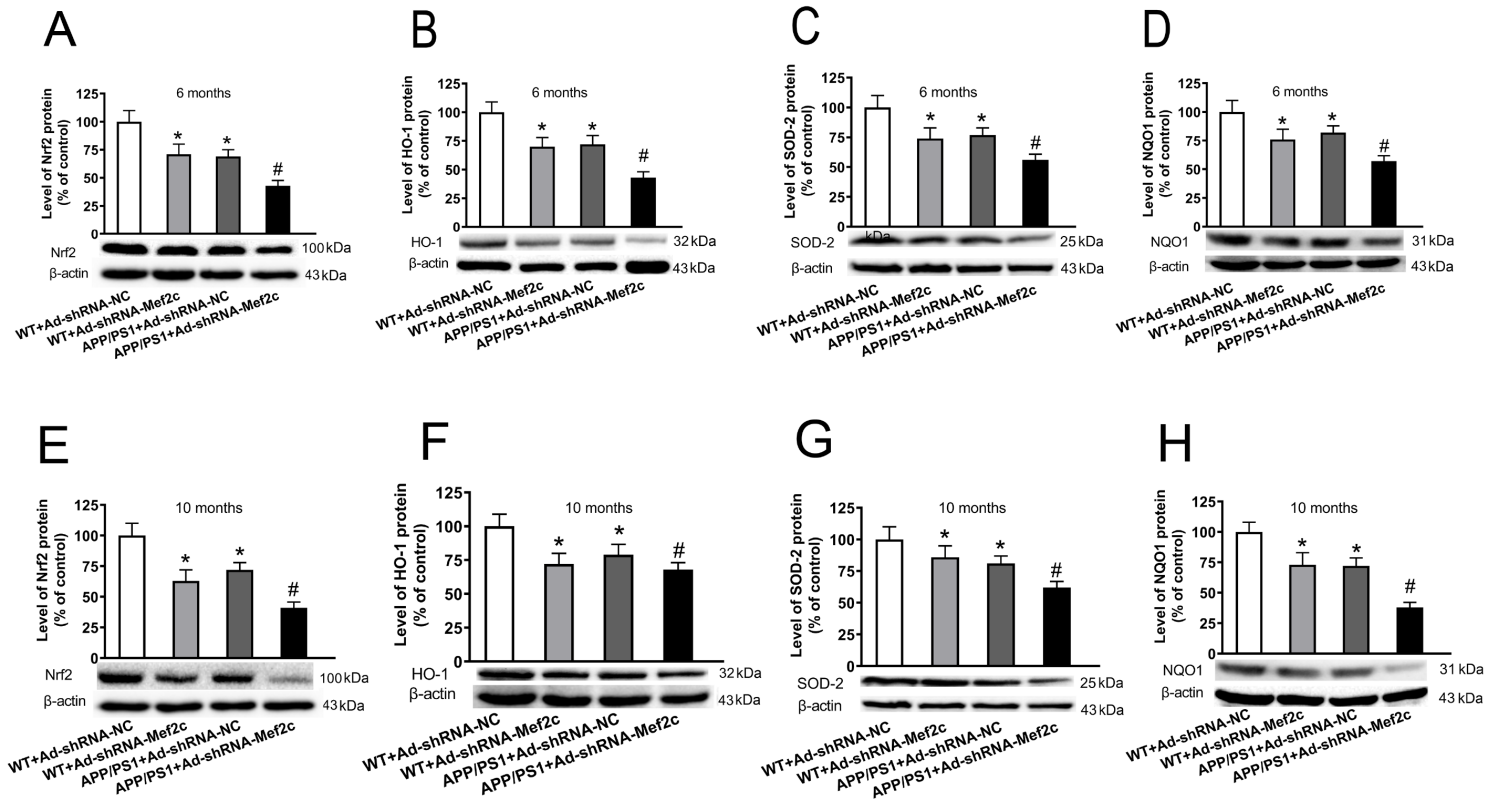
Figure7 **Knockdown of Mef2c reduced the expression of Nrf2-antioxidant response element pathway-related proteins in the cortex of APP/PS1 mice**(A,E) The level of Nrf2 in the cortex of 6- and 10-month-old mice was detected by western blot anlaysis. (B,F) The level of heme oxygenase 1 in the cortex of 6- and 10-month-old mice was detected by western blot analysis. (C,G) The level of superoxide dismutase 2 in the cortex of 6- and 10-month-old mice was detected by western blot analysis. (D,H) The level of NADPH quinone dehydrogenase 1 in the cortex of 6- and 10-month-old mice was detected by western blot analysis. C57 WT mice were injected with Ad-shRNA-NC (WT+Ad-shRNA-NC) or Ad-shRNA-Mef2c (WT+Ad-shRNA-Mef2c). APP/PS1 mice were injected with Ad-shRNA-NC (APP/PS1+Ad-shRNA-NC) or Ad-shRNA-Mef2c (APP/PS1+Ad-shRNA-Mef2c). Data are presented as the mean±standard deviation. *P<0.05 vs WT+Ad-shRNA-NC; #P<0.05 vs APP/PS1+Ad-shRNA-NC.

**Figure 8:**
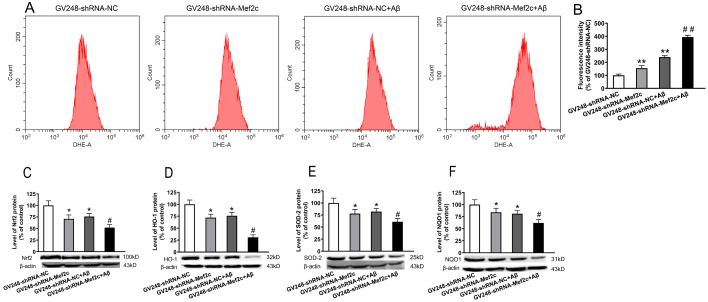
Figure8 **Knockdown of MEF2C increased the ROS level and reduced the expression of Nrf2-ARE pathway-related proteins in SH-SY5Y cells treated with AβO**(A) The level of ROS in cells was detected by flow cytometry. (B) Mean DHE-A for each group of cells. (C) The level of Nrf2 in SH-SY5Y cells was detected by western blot analysis. (D) The level of heme oxygenase 1 in SH-SY5Y cells was detected by western blot analysis. (E) The level of superoxide dismutase 2 in SH-SY5Y cells was detected by western blot analysis. (F) The level of NADPH quinone dehydrogenase 1 in SH-SY5Y cells was detected by western blot analysis. SH-SY5Y cells were transfected with GV248-shRNA-NC (GV248-shRNA-NC) or GV248-shRNA-Mef2c (GV248-shRNA-Mef2c). SH-SY5Y cells were transfected with GV248-shRNA-NC and exposed to AβO (GV248-shRNA-NC+Aβ). SH-SY5Y cells were transfected with GV248-shRNA-Mef2c and exposed to AβO (GV248-shRNA-Mef2c+Aβ). Data are presented as the mean±standard deviation. *P<0.05, **P<0.01 vs GV248-shRNA-NC; #P<0.05, ##P<0.01 vs GV248-shRNA-NC+Aβ.

The Nrf2-ARE signaling pathway plays an important role in regulating oxidative stress, and it is also a potential therapeutic target for treating of AD. We investigated the effects of Mef2c knockdown on Nrf2-ARE signaling pathway in the hippocampal neurons and the ROS level in SH-SY5Y cells. The results showed that in 6- and 10-month-old mice, compared with those in the WT+Ad-shRNA-NC group, the expression levels of Nrf2 ( Figure 7A,E), HO-1 ( Figure 7B,F), SOD2 ( Figure 7C,G) and NQO1 ( Figure 7D,H) in the WT+Ad-shRNA-Mef2c and APP/PS1+Ad-shRNA-NC groups were decreased. Compared with those in the APP/PS1+Ad-shRNA-NC group, the protein expression levels of Nrf2 ( Figure 7A,E), HO-1 ( Figure 7B,F), SOD2 ( Figure 7C,G), NQO1 ( Figure 7D,H) were decreased in the APP/PS1+Ad-shRNA-Mef2c group. The results are similar in SH-SY5Y cells treated with AβO. Aβ increased the ROS level ( Figure 8A,B), decreased the expression of Nrf2 ( Figure 8C), HO-1 ( Figure 8D), SOD2 ( Figure 8E), and NQO1 ( Figure 8F), while knock-down of MEF2C enhanced such effects of Aβ. These data indicate that MEF2C knockdown may decrease the antioxidant capacity by reducing the level of Nrf2-ARE pathway proteins.

### MEF2C silencing exacerbated oxidative stress

To study the effect of MEF2C on the level of oxidative stress in the brain of APP/PS1_DT mice, we measured the content of MDA and SOD activity in the brain cortex tissue. Compared with that in the WT+Ad-shRNA-NC group, the MDA content was increased, while the SOD activity was decreased in the WT+Ad-shRNA-Mef2c and APP/PS1+Ad-shRNA-NC groups. Compared with that in the APP/PS1+Ad-shRNA-NC group, the MDA content was increased, while the SOD activity was reduced in the APP/PS1+Ad-shRNA-Mef2c group ( Table 2). These data indicate that the level of oxidative stress in the brain tissue of APP/PS1-DT mice was increased, and silencing of Mef2c further aggravated the level of oxidative stress, which may be related to the mechanism of AD.

**Table:** **Table2** The MDA and SOD activities in the cortex of 6- and 10-month-old mice

Group	MDA (nmol/mg protein)	SOD (U/mg protein)
	6-month-old	10-month-old	6-month-old	10-month-old
WT+Ad-shRNA-NC	2.035±0.03	2.105±0.036	2.24±0.04	2.4 ±0.10
WT+Ad-shRNA-MEF2C	3.42±0.02*	2.66±0.11*	1.96±0.049*	1.68±0.02*
APP/PS1+Ad-shRNA-NC	3.21±0.04*	3.11±0.049*	1.82±0.061*	1.66±0.055*
APP/PS1+Ad-shRNA-MEF2C	3.72±0.012 ^#^	3.83±0.065 ^#^	1.18±0.18 ^#^	0.87±0.12 ^#^

* *P*<0.05 vs WT+Ad-shRNA-NC; ^#^ *P*<0.05 vs APP/PS1+Ad-shRNA-NC.

### Knockdown of Mef2c reduced the expression levels of synapse-associated proteins in the cortex of APP/PS1 mice and SH-SY5Y cells treated with AβO

**Figure 9:**
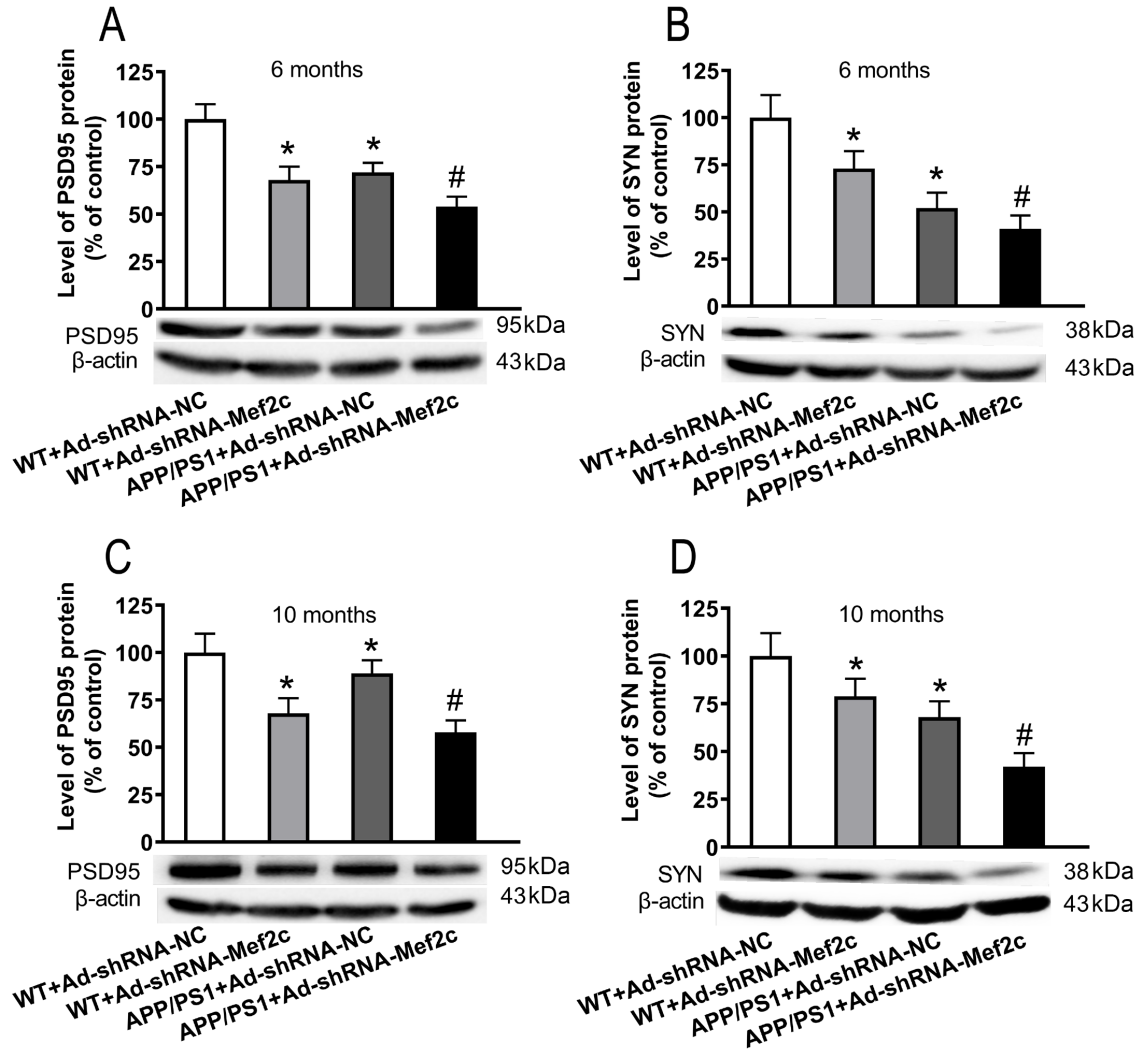
Figure9 **Knockdown of Mef2c reduced synapse-associated proteins in the cortex of APP/PS1 mice**(A,C) The level of PSD95 was detected in the cortex of 6- and 10-month-old mice by western blot analysis. (B,D) The level of SYN was detected in the cortex of 6- and 10-month-old mice by western blot analysis. C57 WT mice were injected with Ad-shRNA-NC (WT+Ad-shRNA-NC) or Ad-shRNA-Mef2c (WT+Ad-shRNA-Mef2c). APP/PS1 mice were injected with Ad-shRNA-NC (APP/PS1+Ad-shRNA) or Ad-shRNA-Mef2c (APP/PS1+Ad-shRNA-Mef2c). Data are presented as the mean±standard deviation. *P<0.05 vs WT+Ad-shRNA-NC; #P<0.05 vs APP/PS1+Ad-shRNA-NC.

**Figure 10:**
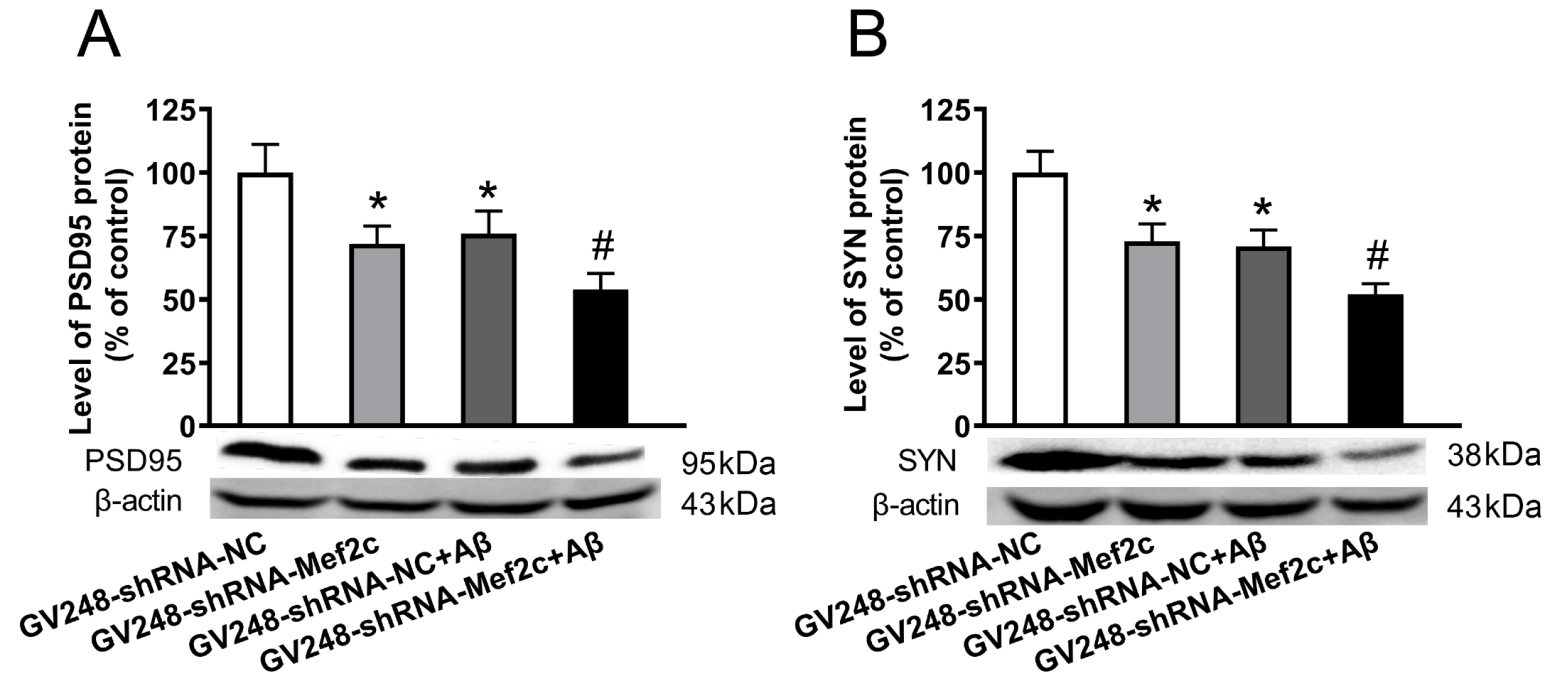
Figure10 **Knockdown of MEF2C reduced the level of synapse-associated proteins in SH-SY5Y cells treated with AβO**(A) The protein level of PSD95 in cells was detected by western blot analysis. (B) The protein level of SYN in cells weas detected by western blot analysis. SH-SY5Y cells were transfected with GV248-shRNA-NC (GV248-shRNA-NC) or GV248-shRNA-Mef2c (GV248-shRNA-Mef2c). SH-SY5Y cells were transfected with GV248-shRNA-NC and exposed to AβO (GV248-shRNA-NC+Aβ). SH-SY5Y cells were transfected with GV248-shRNA-Mef2c and exposed to AβO (GV248-shRNA-Mef2c+Aβ). Data are presented as the mean±standard deviation. *P<0.05 vs GV248-shRNA-NC; #P<0.05 vs GV248-shRNA-NC+Aβ.

To explore whether knockdown of Mef2c could affect the levels of synaptic-associated proteins in the brain of APP/PS1_DT mice and SH-SY5Y cells treated with AβO, western blot analysis was used. As shown in Figure 9, compared with that in the WT+Ad-shRNA-NC group, the protein expressions of PSD95 ( Figure 9A,C) and SYN ( Figure 9B,D) were decreased in the WT+Ad-shRNA-Mef2c and APP+Ad-shRNA-NC groups. Compared with that in the APP/PS1+Ad-shRNA-NC group, the expressions of PSD95 ( Figure 9A,C) and SYN ( Figure 9B,D) were reduced in the APP/PS1+Ad-shRNA-Mef2c group. The results were same in SH-SY5Y cells treated with AβO, as Aβ decreased the protein levels of PSD95 ( Figure 10A) and SYN ( Figure 10B), while knockdown of MEF2C enhanced such effects of Aβ. These data indicate that MEF2C silencing aggravates the synaptic dysfunction both *in vivo* and *in vitro*.

## Discussion

It has been previously reported that the mRNA expression of MEF2C in the blood of patients with AD is reduced [14]; however, changes in the brain of patients with AD have not been reported to date. In this study we found that compared with the normal control group, the expression of MEF2C in the temporal and frontal lobes of patients with AD was reduced, and it was hardly expressed in the hippocampus. A large quantity of Aβ was scattered in the temporal lobe, frontal lobe, and hippocampus of patients with AD. Pearson correlation analysis showed that the levels of MEF2C and Aβ were negatively correlated in different brain regions of patients with AD. The above results indicated that the expression of MEF2C was reduced in the cortex of patients with AD, which might due to the high level of Aβ aggregation in the cortex of patients with AD. MEF2C might participate in Aβ deposition or reduce Aβ neurotoxicity in the brain of patients with AD.

The ‘amyloid cascade hypothesis’ suggests that senile plaques produced by the aggregation of Aβ lead to the occurrence of AD [28]. APP in the brain undergoes β and γ-secretase-mediated hydrolysis of amyloid metabolic pathways, α and γ-secretase-mediated hydrolysis of non-amyloid metabolic pathways. The APP produced by hydrolysis of the amyloid pathways produces a polypeptide composed of 39–43 amino acid residues, which is neurotoxic [29]. Among them, β-secretase-related protein BACE1 promotes the production of Aβ, while its homologue BACE2 inhibits the production of Aβ [30]. This study further detected the expression of Mef2c and the deposition of Aβ in the brain of APP/PS1 mice. It was also found that Mef2c was mainly expressed in the cortex of mice and the expression level was reduced; meanwhile Aβ was abundant and scattered, the level of BACE1 was increased, and the level of BACE2 was decreased in the cortex of APP/PS1 mice. It has been reported that MEF2C is a regulator of the process of APP hydrolysis to produce Aβ [31]. In agreement with this, we found that the deposition of Aβ was increased, which might be related to the up-regulation of BACE1 and down-regulation of BACE2 in the cortex of APP/PS1 mice with Mef2c knockdown. Meanwhile, the learning and memory ability was significantly reduced in the APP/PS1 mice with Mer2c knockdown at 6- and 10-month-old. As Aβ plays a very important role in the cognitive dysfunction in AD, knockdown of Mef2c was found to further worsen the learning and memory ability of APP/PS1 mice, which might be related to reduced deposition of Aβ in the brain. However, further studies are necessary to explore the mechanism of this phenomenon.

Many previous studies have confirmed that OS is an important pathological feature in AD pathogenesis [ 30, 32]. The deposition of Aβ can cause oxidative stress damage to nerve cells and OS promotes the production and aggregation of Aβ, which forms a vicious circle between them [33]. The Nrf2-ARE signal pathway plays an important role in maintaining the balance between oxidation and anti-oxidation in the body [18]. In addition, the occurrence of OS will increase oxygen free radicals, which will also damage neurons and accelerate their aging, leading to learning and memory disorders. Nrf2 is a major transcription factor and can regulate genes related to OS, and its promoter contains antioxidant response elements [34]. Nrf2 structural and functional changes were found in most neurodegenerative diseases, such as AD [35], Parkinson′s disease (PD) [36] and amyotrophic lateral sclerosis [37]. Nrf2 protects the body from oxidative stress damage by up-regulating antioxidant defense pathway activation, inhibiting inflammation and maintaining protein homeostasis. Nrf2 combines with ARE to regulate the activation of important antioxidant enzymes such as NQO1, SOD and HO-1, which in turn regulates the level of OS in the body. Due to its role in regulating OS, the Nrf2-ARE pathway was recommended as a target for the treatment of AD in recent years [38]. Several studies have reported that the level of Nrf2 is significantly reduced in AD models, and the activity of Nrf2-ARE signal pathway is also reduced. As MEF2C can indirectly activate Nrf2 according to KEGG analysis, this study further analyzed whether MEF2C could regulate the Nrf2 signal pathway.

The results showed that: the content of MDA was increased, while the activity of SOD was decreased in the APP/PS1 mice cortex; the ROS level was increased in SH-SY5Y cells treated with AβO; the expression of Nrf2-ARE signal pathway-associated proteins such as Nrf2, HO-1, SOD2 and NQO1 in APP/PS1 mouse cortex and in SH-SY5Y cells treated with Aβ were reduced significantly, which was consistent with previous reports, indicating that Aβ-induced OS was a main mechanism in the pathogenesis of AD. With the knockdown of Mef2c in APP/PS1 mice with the knockdown of Mef2c, the Aβ-induced OS was more serious, the content of MDA in brain tissue was significantly increased, and SOD activity was decreased compared with those in APP/PS1 mice. Meanwhile, with the knockdown of MEF2C in SH-SY5Y cells, the ROS level was increased compared with that in SH-SY5Y cells treated with AβO. The downregulation of MEF2C level further decreased the levels of Nrf2-ARE signal pathway proteins, compared with the corresponding controls (APP/PS1 mice and SH-SY5Y cells treated with AβO). The Nrf2-ARE signal pathway could regulate the activation of important antioxidant enzymes such as NQO1, SOD and HO-1, which in turn could regulate the level of OS [19]. MEF2C might indirectly affect the OS level by regulating the Nrf2-ARE signal pathway.

By crossing NRF2 ^−/−^ mice and APP/PS1 mice, Joshi *et al*. [39] observed that, compared with those in APP/PS1 mice, the levels of Aβ fragments, APP fragments and total APP were increased in neurons of APP/PS1/NRF2 ^−/−^ mice. Nrf2-ARE pathway could negatively regulate BACE1 to affect the production of Aβ [40]. Our results also showed that knockdown of Mef2c significantly increased the deposition of Aβ in the cortex, and the expression of BACE1 was increased, which further indicated that MEF2C might reduce the production of Aβ by upregulating the Nrf2 pathway.

Many studies have shown that the Aβ deposition leads to impaired synaptic integrity and function, which is considered to be the vital cause of cognitive decline in AD [41]. Synaptic function is largely correlated with synaptic proteins, including pre- and post-synaptic membrane proteins and vesicle-associated proteins. Our previous and present studies all indicated that Aβ accumulation down-regulates the synaptic-associated proteins levels, including SYN, PSD95, and DYN1 in APP/PS1_DT mice model in an age-dependent manner, and then affects the synaptic function [42]. Nrf2-ARE signal pathway, as an important anti-OS pathway in the body, plays an important role in regulating the learning and memory functions of patients with AD. Up-regulating the Nrf2 level in brain of 9-month-old APP/PS1 mice and an AD mouse model with PS1 gene mutation all led to improved spatial learning and memory [ 22, 43]. While knockdown of Nrf2 increased the loss of synapse-related proteins in the SAMP8 AD model brain [26]. Therefore, it was speculated that the Nrf2-ARE pathway might be inhibited in the cortex of APP/PS1 mice, which could reduce the expressions of PSD95 and SYN. Our results showed that knockdown of Mef2c in the cortex of APP/PS1 mice reduced their learning and memory abilities, and inhibited the expressions of PSD95 and SYN. We also found that the apoptosis rate was increased, the level of BAX was increased, and BCL-2 was decreased in both the cortex of APP/PS1 mice and SH-SY5Y cells treated with Aβ. Down-regulation of MEF2C enhanced such effects. It has been reported that MEF2C plays an important role in promoting neuronal survival and inhibiting apoptosis of neurons in the nervous system [44]. MEF2C plays an important role in preventing APP-mediated apoptosis of nerve cells [45]. Our results showed that the mechanism might involve the inhibition of the Nrf2-ARE signal pathway by knocking down MEF2C, which further increased the deposition of Aβ and decreased the expressions of synapse-related proteins.

In summary, in this study we found that the expression of MEF2C is decreased in the brain of AD patients, which might be one of the reasons or results for the deposition of Aβ in AD and aggravation of its neurotoxicity. Knockdown of MEF2C could inhibit the Nrf2-ARE signal pathway, thus regulating the level of APP metabolic enzymes, accelerating the production and aggregation of Aβ, increasing the level of OS, and damaging synaptic plasticity, which ultimately leads to increased apoptosis, reduced ability of learning and memory, and aggravated damage to nerve cells. Therefore, MEF2C might play a neuroprotective role in AD by activating the Nrf2-ARE signal pathway. It is hopeful that this research may shed some light on the underlying pathogenesis of AD.

## Supporting information

322FigS3

322FigS1

322FigS2

322TableS1

## Supplementary Data

Supplementary Data is available at *Acta Biochimica et Biphysica Sinica* online.
