# Supplementary figures and images for: MEF2C ameliorates learning, memory, and molecular pathological changes in Alzheimer’s disease in vivo and in vitro : Neuroprotective effects of MEF2C
Source: Acta Biochim Biophys Sin (Shanghai). 2021 Dec 28;54(1):77–90. doi: 10.3724/abbs.2021012 (PMC9909301; doi:10.3724/abbs.2021012)

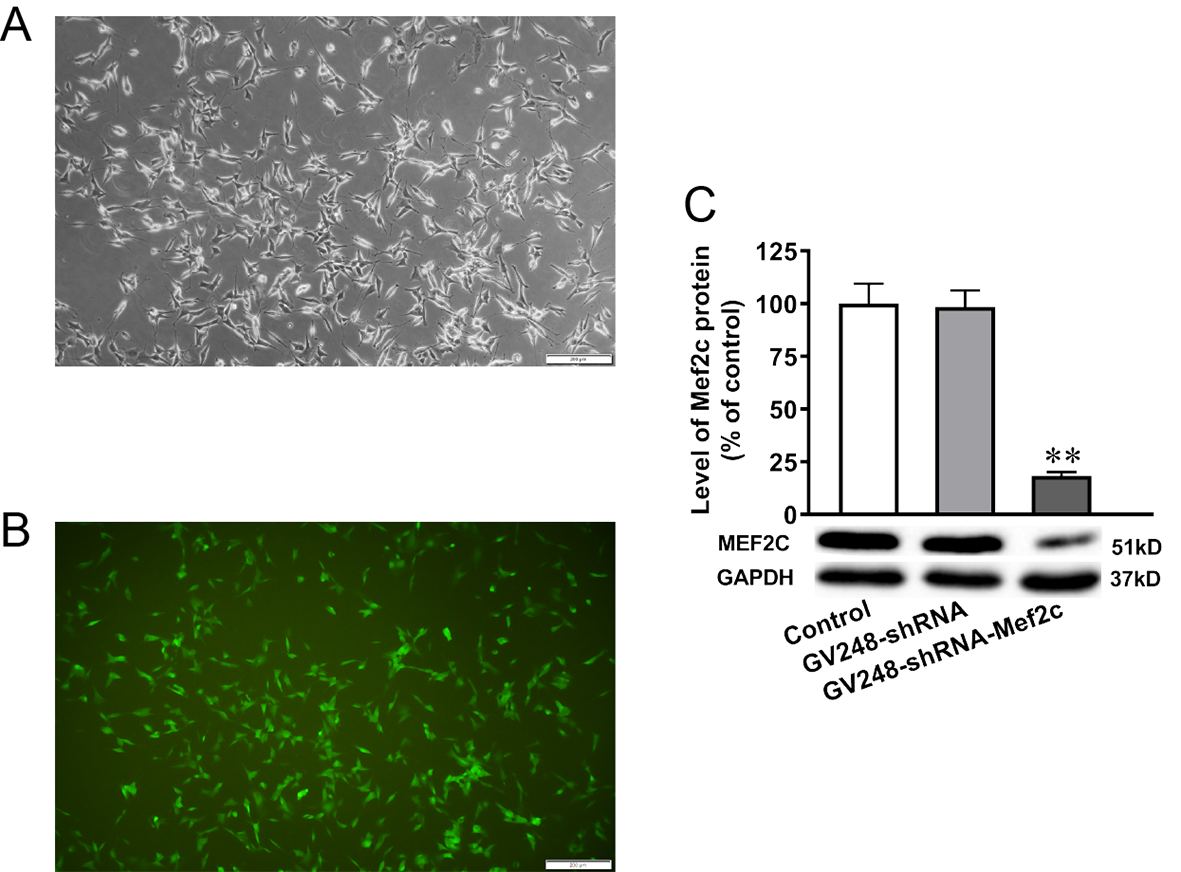

Supplement: 322FigS3 [file 322FigS3.tif]

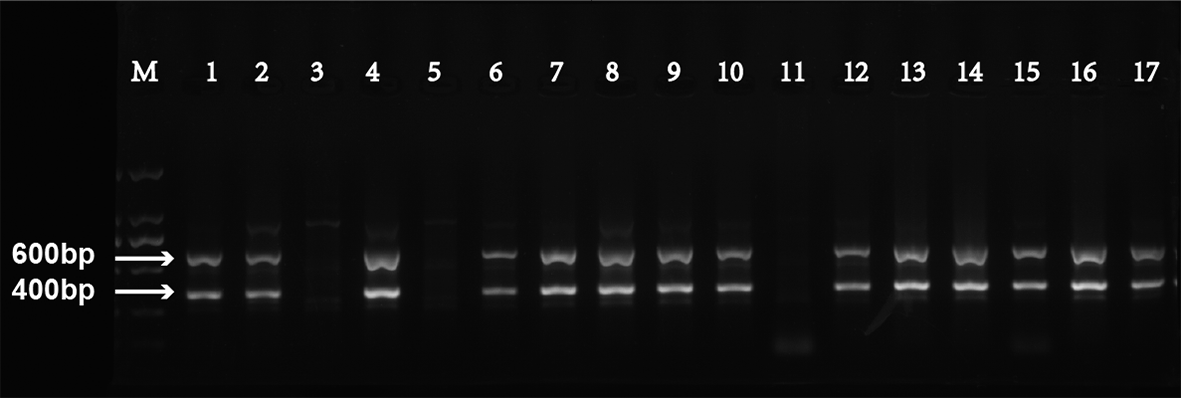

Supplement: 322FigS1 [file 322FigS1.tif]

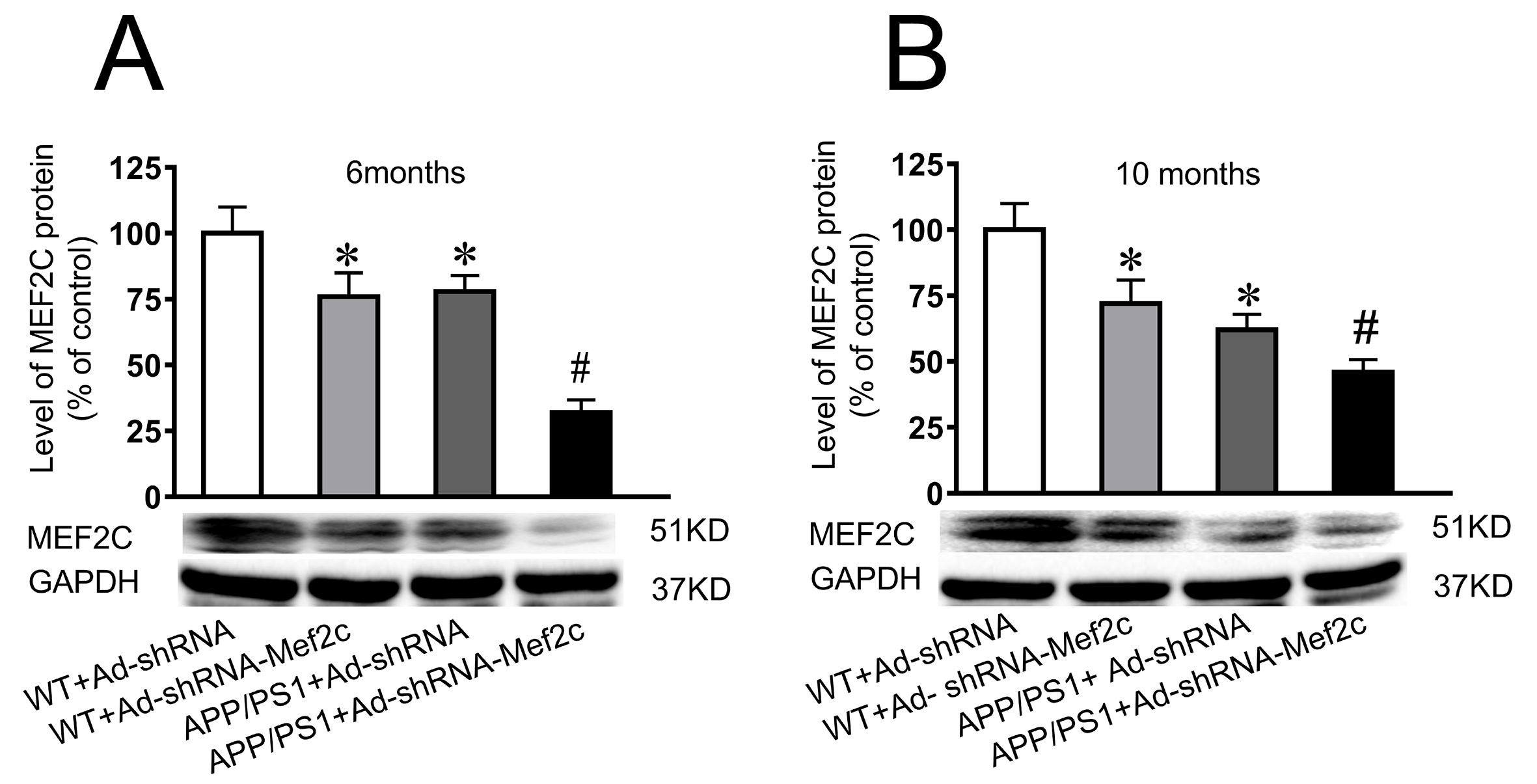

Supplement: 322FigS2 [file 322FigS2.tif]
