# Supplementary material for: MEF2C ameliorates learning, memory, and molecular pathological changes in Alzheimer’s disease in vivo and in vitro : Neuroprotective effects of MEF2C
Source: Acta Biochim Biophys Sin (Shanghai). 2021 Dec 28;54(1):77–90. doi: 10.3724/abbs.2021012 (PMC9909301; doi:10.3724/abbs.2021012)
Supplement: 322TableS1 [file 322TableS1.doc]

**Supplementary Table S1. Sequences of primers** used for identification of APP/PS1 mice

| Gene | Primer sequence (5′→3′) |
| --- | --- |
| *APP*  *PS1* | Forward: GACTGACCACTCGACCAGGTTCTG  Reverse: CTTGTAAGTTGGATTCTCATATCCG  Forward: AATAGAGAACGGCAGGAGCA  Reverse: GCCATGAGGGCACTAATCAT |
